# Rational design, cognition and neuropathology evaluation of QTC-4-MeOBnE in a streptozotocin-induced mouse model of sporadic Alzheimer’s disease

**DOI:** 10.1038/s41598-019-43532-9

**Published:** 2019-05-13

**Authors:** Mariana G. Fronza, Rodolfo Baldinotti, Maria Clara Martins, Bruna Goldani, Bianca Thaís Dalberto, Frederico Schmitt Kremer, Karine Begnini, Luciano da Silva Pinto, Eder João Lenardão, Fabiana K. Seixas, Tiago Collares, Diego Alves, Lucielli Savegnago

**Affiliations:** 1 Research Group on Neurobiotechnology - GPN, CDTec, Federal University of Pelotas, UFPel, Pelotas, RS Brazil; 2 Laboratory of Clean Organic Synthesis - LASOL, CCQFA, Federal University of Pelotas, Pelotas, RS Brazil; 3 Laboratory of Bioinformatics and Proteomics - BIOPRO-LAB, CDTec, Federal University of Pelotas, UFPel, Pelotas, RS Brazil; 4 Oncology Research Group - GPO, CDTec, Federal University of Pelotas, UFPel, Pelotas, RS Brazil

**Keywords:** Pharmacology, Pharmacology

## Abstract

Alzheimer’s disease (AD) is a multifactorial pathology characterized by amyloid deposits, neurofibrillary formation, oxidative stress and cholinergic system dysfunction. In this sense, here we report the rational design of a multi-target directed ligand (MTDL) for AD based on virtual screening and bioinformatic analyses, exploring the molecular targets β-secretase (BACE-1), glycogen synthase kinase-3β (GSK-3β) and acetylcholinesterase (AChE). After this screening, the compound with higher molecular docking affinity was selected, the 1-(7-chloroquinolin-4-yl)-N-(4-methoxybenzyl)-5-methyl-1H-1,2,3-triazole-4 carboxamide(QTC-4-MeOBnE). To further our studies, the protective effect of QTC-4-MeOBnE (0.1 and 1 mg/kg for 20 days) on STZ-induced sporadic AD mice was determined. QTC-4-MeOBnE pretreatment attenuated cognitive and memory deficit induced by STZ in an object recognition test, Y-maze, social recognition test and step-down passive avoidance. The mechanisms underlying this action might be attributed to the reduction of lipid peroxidation and reactive species formation in the prefrontal cortex and hippocampus of mice submitted to STZ. In addition, QTC-4-MeOBnE pretreatment abolished the up-regulation of AChE activity and the overexpression of *GSK* 3β and genes involved in amyloid cascade such as BACE-1, protein precursor amyloid, у-secretase, induced by STZ. Moreover, toxicological parameters were not modified by QTC-4-MeOBnE chronic treatment. This evidence suggests that QTC-4-MeOBnE exerts its therapeutic effect through multiple pathways involved in AD.

## Introduction

Alzheimer Disease (AD) is the most common cause of dementia, with a prevalence of more than 50 million cases in 2015^1^. AD is a neurodegenerative disease, characterized by progressive memory loss and impaired cognitive ability. The predominant pathological hallmarks are the deposition of β-amyloid (Aβ) in senile plaques and neurofibrillary tangles (NFT) composed by hyperphosphorylated TAU^2^. These alterations occur together with extensive oxidative stress, chronic neuroinflammation, excitotoxicity, and mitochondrial dysfunction. Together, these events are considered the main effectors of synaptic dysfunction and the neurodegenerative progression^3,4^.

From this perspective, AD is the major source of unmet medical needs in neurology with high rate of failed clinical trials^5^. These failures can be attributed, in part, to the selectivity of the tested drugs against a single target, while AD is a complex and multifactorial disease^6^. At the same time, several biological targets for potential therapeutics have been identified, suggesting the research strategy of development, a multi-target approach. This concept has recently been illustrated by the “multitarget-directed ligands” (MTDL’s), consisting in a single molecule which can modulate different relevant therapeutic targets directly^7^.

Within this scope, β-secretase (BACE-1) is the rate limiting step of Aβ-42 production, which is responsible for the first cleavage of amyloid precursor protein (APP)^8^. Aβ induces inflammation as well as, oxidative stress by exacerbating the production of reactive species (RS) and mitochondrial dysfunction and morphology^9^. Oxidative stress and lipid peroxidation can directly activate GSK-3β, mainly kinase responsible for TAU hyperphosphorylation and aggregation^10^. Furthermore, the accumulation of hyperphosphorylated TAU also contributes to Aβ neurotoxicity^11^.

All these events have a crucial role in neurodegeneration and synaptic loss, which is the main correlate of AD cognitive decline^12^. The cholinergic synapses are selectively vulnerable to Aβ oligomer toxicity, being drastically affected by neurodegeneration^13^. In addition, structural studies have pointed to the dual inhibition of AChE (catalytic active site (CAS) and peripheral anionic site (PAS) that can reduce both the cholinergic deficit and Aβ deposition^14^.

It is considered that this evidence supports the idea that BACE-1, GSK-3β, AChE and oxidative stress are key components of a vicious circle, crucial in the onset and progression of AD pathogenesis. In this context here we report the rational design of an MTDL compound based on bioinformatics analyses exploring the therapeutic targets such as BACE-1, GSK-3β, AChE and its pharmacokinetic predicted profile. The biological evaluation was performed in a mice model of sporadic late-onset AD induced by intracerebroventricular (ICV) injection of streptozotocin (STZ). This study focused upon the capacity of a compound to prevent learning and memory impairment, and its beneficial effect on oxidative damage, synaptic integrity, AChE activity, expression of amyloidogenic molecular biomarkers and GSK-3β, as well as the renal and hepatotoxicity caused by sub-chronic compound administration.

## Results

### QTC-4-MeOBnE is the result of virtual screening

The central pharmacological target of this study was BACE-1 inhibitors, which are considered a potential treatment for AD, due to their ability to reduce Aβ deposition. However, one of the limiting characteristics of these drugs is the reduced oral bioavailability and the low capacity to cross the BBB. Therefore, we performed a virtual screening based on drug-like properties (molecular weight <500 KDa, Log P, number of hydrogen donors (<5) and acceptors (<10)) to avoid poor pharmacokinetics. This chemical scaffold was utilized to create 79 molecules whose binding affinity to BACE-1 was individually evaluated by molecular docking.

**Figure 1:**
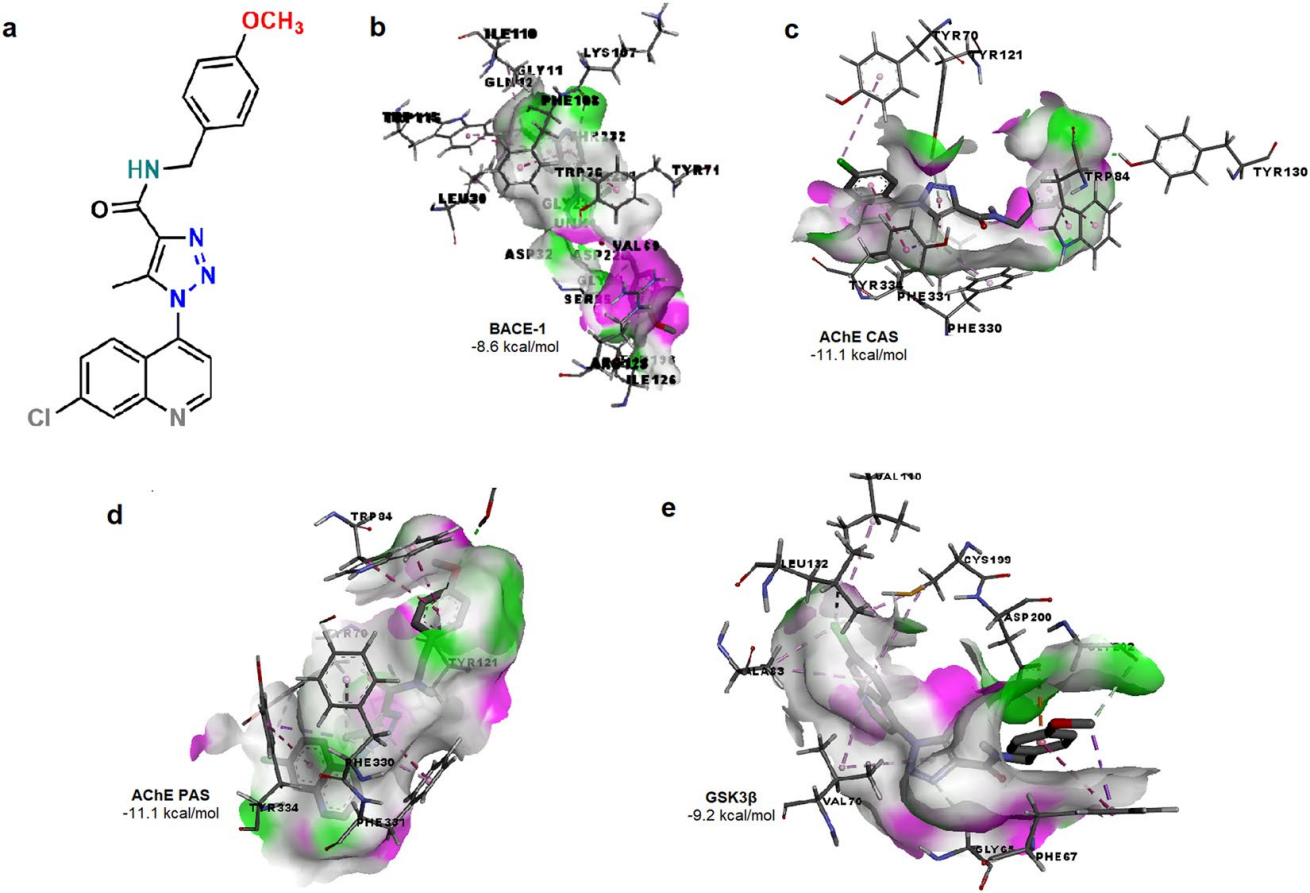
Chemical structure (**a**) and the predicted binding affinity with ligand-protein interaction on (**b**) *β* – secretase (BACE-1), (**c**) acetylcholinesterase (AChE) catalytic active site (CAS), (**d**) AChE peripheral anionic site (PAS), (**e**) Glycogen synthase kinase 3*β* (*GSK3 β*) of 1-(7-chloroquinolin-4-yl)-*N*-(4-methoxybenzyl)-5-methyl-1*H*-1,2,3-triazole-4-carboxamide (QTC-4-MeOBnE).

The molecule with the best docking score on BACE-1 was 1-(7-choloroquinolin-4-yl)-N-(4-methoxybenzyl)-5-methyl-1H-1,2,3-triazole-4 carboxamide (QTC-4-MeOBnE) (Fig. 1a) that possessed a free binding energy of −8.6 kcal/mol. The molecule interacts with the catalytic aspartate dyad, Asp32 and Asp228 (Fig. 1b). Further, QTC-4-MeOBnE interacts with residues belonging to large hydrophobic pockets (S1, S3, S2), which are key regions of BACE inhibition^15^. Our results showed interactions with residues that comprise the hydrophobic cleft of S1 and S3: Trp115, Leu30, Ile 110 and Phe 108, with the main chain of S3: Gln12, Gly11 Gly230 and Thr232 as well as with residues of S2 pocket: Val69 and Ile126.

Multiple clinical trials suggested that BACE-1 inhibitors attenuate Aβ accumulation but fail to improve dementia. Thus, AChE inhibitors, currently approved drugs to treat AD, have been shown to reduce cognitive manifestations, without delaying or halting the progression of the disease^16^.Thus, we evaluated the QTC-4-MeOBnE binding affinity with AChE on CAS and on PAS. CAS is composed of Trp84, Tyr130, Phe330 and Phe331. This motif is responsible for binding the substrate^17^. QTC-4-MeOBnE had a docking score of −11.1 kcal/mol and in this position, interacted with the entire site: Trp84, Tyr130, Phe330, Phe331 and with an additional hydrogen bond with Tyr121 (Fig. 1c). PAS is located at the entry to the active site gorge and is responsible for extra activities, as induce Aβ oligomerization. This site consists of five amino acids: Tyr70, Asp72, Tyr121, Trp279 and Tyr334^17,18^. Our results indicate that QTC-4-MeOBnE is a dual binding site cholinesterase inhibitor, because in this position, the compound had a docking score of −11.1 kcal/mol. As can be seen in Fig. 1d, the compound interacts with Tyr 121, Tyr335, Tyr70 and continues to form a hydrogen bond with Tyr130.

The majority of tau-based therapeutic strategies have focused on modulating tau hyperphosphorylation to reduce the formation of NFT. As GSK-3β is the main kinase responsible for the phosphorylation process of TAU, we performed a molecular docking on its ATP binding pocket, in the motif where the inhibitors described interact^19^. QTC-4-MeOBnE had a high docking score value of −9.2 kcal/mol, interacting with residues of ATP binding pocket: Asp200, Val70, Val110, Ala83, Leu 132 and Cys199 (Fig. 1e). Previous studies reported that interactions with Cys199 and Asp 200 are crucial for GSK3β inhibition^20^.

ADMET properties reveal that QTC-4-MeOBnE crosses the blood brain barrier (BBB) possessing a passive blood-brain partitioning (Log BB) of −0,215, and also had 75% of predictive activity in the central nervous system (CNS). Our results show that QTC-4-MeOBnE is probably almost completely absorbed in human intestine and is highly metabolized by CP450 3A4 enzyme. The compound has low oral toxicity, probably with a DL50 of 500–5000 mg/kg.

### QTC-4-MeOBnE protects memory and cognitive impairment induced by ICV injection of STZ

**Figure 2:**
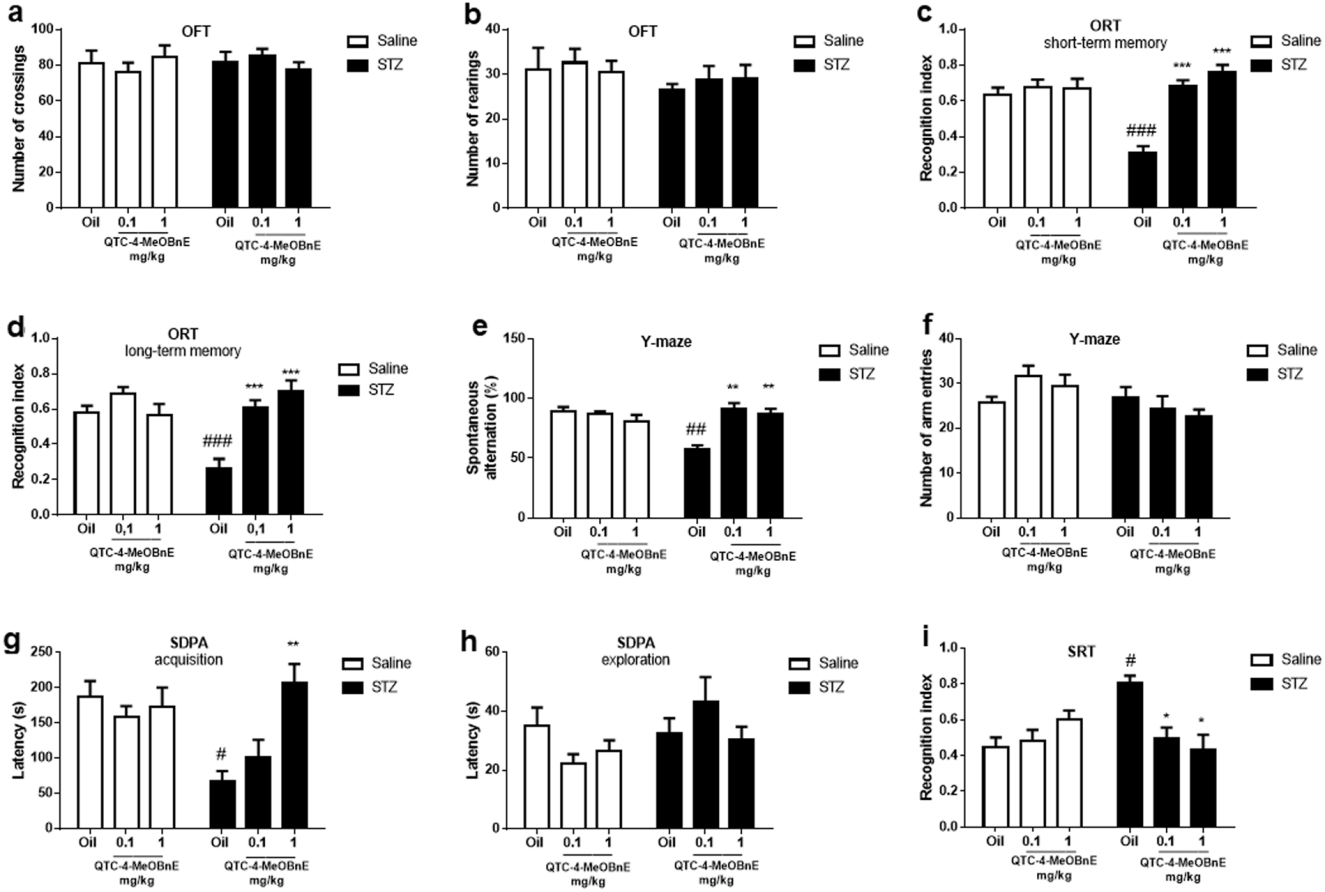
Effect of 20 days pre-treatment with QTC-4-MeOBnE (0.1 and 1 mg/kg intragastrically (i.g.) on behavioral tests of mice submitted to intracerebroventricular (ICV) infusion of streptozotocin (STZ) (3 mg/kg) or saline. Number of (**a**) crossings and (**b**) rearings in the Open Field Test (OFT); Recognition index on (**c**) short- term memory and (**d**) long-term memory evaluated in the Object Recognition Test (ORT); (**e**) spontaneous alternation (%) and (**f**) number of entries in the Y-maze test; Latency time of (**g**) acquisition and (**h**) exploration in Step-Down Passive Avoidance (SDPA); (**i**) Social recognition index on Social Recognition Test (SRT); Results are expressed as mean ± SEM. (n = 6–8) ^#^p < 0.05, ^##^p < 0.01 and ^###^p < 0.001 when compared with the sham group. *p < 0.05, **p < 0.01 and ***p < 0.001 when compared to the STZ group (two-way ANOVA followed by Newman-Keuls test).

Due the promising characteristics, the effect of pretreatment with QTC-4-MeOBnE (0.1 and 1 mg/kg for 20 days) was evaluated on a STZ-induced mouse model of sporadic AD. First, mice were evaluated in Open Field Test (OFT) to avoid any false results induced by altered locomotor activity in the other behavioral tests. The two-way ANOVA revealed the absence of statistically significant interaction between STZ x QTC-4-MeOBnE for crossings [F_(2,36_) = 1.078, p = 0.35] (Fig. 2a) and rearings [F_(2,36_) = 0.1577, p = 0.85] (Fig. 2b) demonstrating that treatment with QTC-4-MeOBnE did not induce psychostimulant disturbances.

The short-term memory, in the Object Recognition Test (ORT), was defined as 90 min after the presentation of identical objects. The ORT two-way ANOVA indicated significant interaction of STZ x QTC-4-MeOBnE [F_(2, 36)_ = 12.75, p < 0.001]. STZ (3 mg/kg, 2 days) infusion-induced cognitive deficits (Fig. 2c) demonstrated by a significant reduction in the object recognition index, when compared to the sham group. QTC-4-MeOBnE protected against the memory decline in both doses tested (0.1 and 1 mg/kg) when compared to animals treated only with STZ and vehicle (canola oil).

The same reduction of the recognition index in the ORT of animals treated with STZ was observed in long-term memory, defined in the present study as 24 h after the habituation with identical objects. Two-way ANOVA analyses showed a significant interaction of STZ x QTC-4-MeOBnE [F_(2, 36)_ = 9.911, p < 0.001]. QTC-4-MeOBnE treatment, in both doses tested, was effective to protect against the impaired recognition index compared to mice treated with STZ and canola oil (Fig. 2d).

Two-way ANOVA of Y-maze revealed significant interaction between STZ x QTC-4-MeOBnE for spontaneous alternation [F_(2,30)_ = 12.37, p < 0.001] and no interaction for the number of entries [F_(2,30)_ = 2.186, p = 0.13]. The STZ administration induced a decline in spatial and working memory, otherwise, the treatment with QTC-4-MeOBnE at doses of 0.1 and 1 mg/kg prevented memory impairment, defined by an increasing percentage of spontaneous alternation behavior (Fig. 2e). This increase was independent of number of arm entries, excluding any false positive related to altered locomotor function in this test (Fig. 2f).

The Step-Down Passive Avoidance (SDPA) test is extensively used to evaluate learning and aversive memory through the latency time in stepping down from the platform, after an electric shock^21^. In this study, the two-way ANOVA indicates a statistically significant interaction between STZ x QTC-4-MeOBnE on acquisition latency time [F_(2, 36)_ = 5.958, p = 0.006] and no significance in transfer latency time [F_(2, 36)_ = 1.422, p = 0.25]. STZ induced memory impairment when compared to the sham group and the pretreatment with QTC-4-MeOBnE 1 mg/kg was efficient efficient in preventing this increase (Fig. 2g). This result indicates that only the highest compound dosage can protect against learning deficit through aversive memory formation.

We also evaluated the latency time to step down from the platform in the training phase, defined as exploration time. There was no difference in the transfer latency time among groups, validating the SPDA test and suggesting that these results were not related to animal exploratory activity (Fig. 2h).

The social recognition memory was assessed utilizing the SRT, which simulates the ability of animals to discriminate between familiar and unfamiliar stimuli. SRT Two-way ANOVA demonstrated a significant STZ x QTC-4-MeOBnE interaction [F_(2, 30)_ = 9.422, p < 0.001]. The treatment with QTC-4-MeOBnE, in both tested doses, was efficient in preventing memory decline induced by ICV injection of STZ (Fig. 2i). This result, is represented by an increase of social contact with the juvenile intruder, demonstrating a social memory recognition.

Overall, the treatment with QTC-4-MeOBnE (0.1 and 1 mg/kg) in mice subjected to ICV injection of saline did not present any significant difference between groups in behavioral tests performed. These results suggest that QTC-4-MeOBnE did not present effects of improved cognition *per se*, it just prevented the cognitive impairment induced by STZ.

### Treatment with QTC-4-MeOBnA prevents the oxidative damage induced by STZ

**Figure 3:**
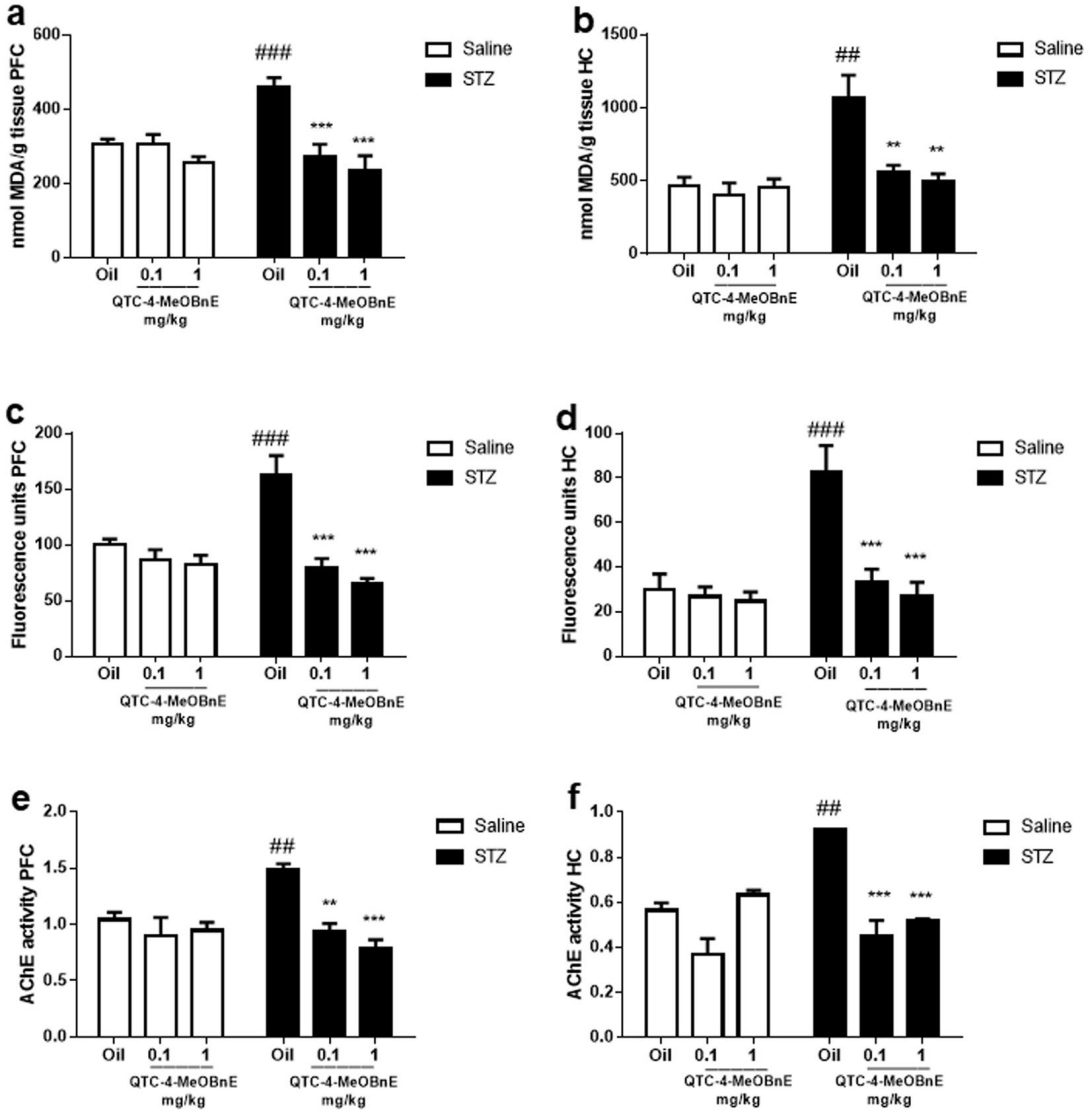
Effect of 20 days pre-treatment with QTC-4-MeOBnE (0.1 and 1 mg/kg i.g.) on oxidative markers and AChE activity of mice submitted to ICV infusion of STZ (3 mg/kg) or saline. TBARS levels in (**a**) Prefrontal Cortex (PFC) and (**b**) Hippocampus (HC); Reactive species in (**c**) PFC and (**d**) HC; AChE activity, given as *nmol/mg protein/min*, in (**e**) PFC and (**f**) HC. Results are expressed as mean ± SEM. (n = 6–8). ^##^p < 0.01 and ^###^p < 0.001 when compared with the control group. **p < 0.01 and ***p < 0.001 when compared to the STZ group (two-way ANOVA followed by Newman-Keuls test).

It has long been established that biomarkers of lipid peroxidation are elevated in the brain of patients with AD, even in the preclinical phase of the disease, when compared with age-matched controls^22^, suggesting an early and relevant involvement of AD and lipid peroxidation. Two-way ANOVA indicated a significant STZ x QTC-4-MeOBnE interaction for prefrontal cortex (PFC) [F_(2,36)_ = 7.355, p = 0.002] and hippocampus (HC) [F_(2,36)_ = 6.006, p = 0.0056]. The ICV injection of STZ increased TBARS levels in both structures, when compared to the sham group (Fig. 3a,b). The lipid peroxidation induced by STZ was significantly prevented by the administration of QTC-4-MeOBnE (0.1 and 1 mg/kg). In groups treated with QTC-4-MeOBnE (0.1 and 1 mg/kg) and injected with saline there was no significant change in lipid peroxidation compared with the sham group.

Lipid peroxidation is often caused by excessive RS production in AD, accompanied by events such as oxidatively modified proteins and nucleic acids^3^. The intracellular RS levels in this study were measured by DCF units of fluorescence. Two-way ANOVA showed a significant STZ x QTC-4-MeOBnE interaction for PFC [F_(2,36)_ = 10.12, p < 0.001] and HC [F_(2,36)_ = 7.853, p < 0.001]. RS levels were raised by the STZ infusion in PFC (Fig. 3c) and HC (Fig. 3d) compared to the sham group. QTC-4-MeOBnE could protect against RS formation by STZ at both doses tested. The animals injected with saline did not show statistical differences between treatment groups.

### QTC-4-MeOBnE treatment prevents STZ-induced increased levels of AChE activity

Some studies have indicated that AChE activity is increased mainly around amyloid plaques and NFT, which can link cholinergic deficit and AD^23^. Two-way ANOVA showed a significant interaction of STZ x QTC-4-MeOBnE in PFC[F_(2,30)_ = 5.651, p = 0.008] and HC [F_(2,30)_ = 14.74, p < 0.001]. STZ was able to increase AChE activity in the PFC and HC of mice when compared with the sham group (Fig. 3e,f respectively). The increase in AChE activity was prevented by treatment with QTC-4-MeOBnE (0.1 and 1 mg/kg) in PFC and HC. However, the compound did not reduce the AChE activity alone probably due to the time between compound administration and mice euthanasia.

### Pre-treatment with QTC-4-MeOBnE regulates amyloid cascade and might reduce the hyperphosphorylation of TAU

**Figure 4:**
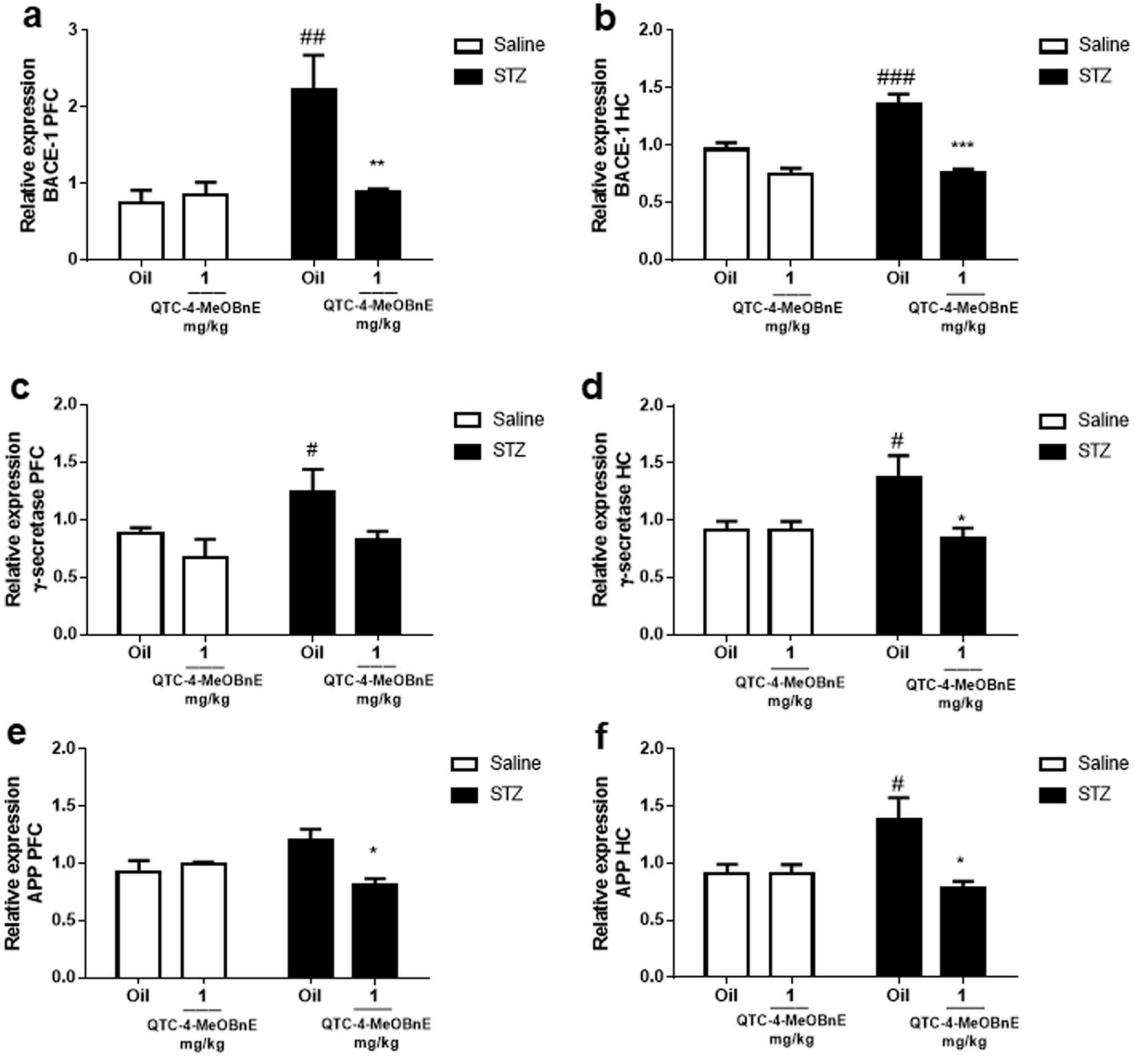
Effect of 20 days pre-treatment with QTC-4-MeOBnE (0.1 and 1 mg/kg i.g.) on amyloid cascade expression of mice submitted to ICV infusion of STZ (3 mg/kg) or saline. BACE-1 expression in (**a**) PFC and (**b**) HC; γ- secretase expression in (**c**) PFC and (**d**) HC; Protein Precursor Amyloid (APP) expression in (**e**) PFC and (**f**) HC. Results are expressed as mean ± SEM. (n = 5–6). ^#^p < 0.05 and ^###^p < 0.001 when compared with the control group. *p < 0.05, **p < 0.01 and ***p < 0.001 when compared with the STZ group (two-way ANOVA followed by Newman-Keuls test).

Since amyloid plaques are the main hallmark of AD, the expressions of APP, BACE-1 and γ- secretase were evaluated. Analyses of two-way ANOVA demonstrated that STZ x QTC-4-MeOBnE interaction in PFC is significant for BACE-1 [F_(1,16)_ = 7.696, p = 0.01], APP [F_(1,16)_ = 8.657, p = 0.010] and the STZ main effect for γ- secretase [F_(1,16)_ = 5.106, p = 0.0382]. As regards the HC analyses revealed a significant STZ x QTC-4-MeOBnE interaction for BACE-1 [F_(1,16)_ = 9.25, p = 0.0078], APP [F_(1,16)_ = 6.348, p = 0.02] and for γ- secretase [F_(1,16)_ = 4.607, p = 0.05]. As expected, STZ injection clearly increased the expression of BACE-1 and γ- secretase in PFC, but not of APP (Fig. 4a,c,e). On the other hand, STZ injection significantly increased the expression of all these enzymes in HC (Fig. 4b,d,f).

The chronic treatment with QTC-4-MeOBnE resulted in a reduction of STZ-induced levels of BACE-1 and APP in both PFC and HC. However, the super expression of γ- secretase was prevented by QTC-4-MeOBnE only in the HC structure.

**Figure 5:**
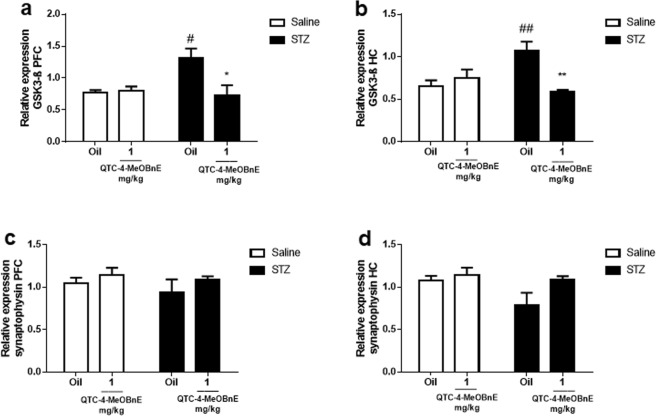
Effect of 20 days pre-treatment with QTC-4-MeOBnE (0.1 and 1 mg/kg i.g.) on GSK3- β and synaptophysin expression of mice submitted to ICV infusion of STZ (3 mg/kg) or saline. GSK3- β expression in (**a**) PFC and (**b**) HC; synaptophysin expression in (**c**) PFC and (**d**) HC. Results are expressed as mean ± SEM. (n = 5–6). ^#^p < 0.05 and ^##^p < 0.01 when compared with the control group. *p < 0.05, **p < 0.01 when compared to STZ group (two-way ANOVA followed by Newman-Keuls test).

The analyses of two-way ANOVA revealed a statistically significant interaction of STZ x QTC-4-MeOBnE of GSK3- β gene expression in PFC [F_(1,16)_ = 6.646, p = 0.02] and HC [F_(1,16)_ = 12.98, p = 0.002]. Interestingly, QTC-4-MeOBnE also prevented the STZ-induced GSK3- β up-regulation in both PFC and HC (Fig. 5a,b). As GSK3- β is the main kinase responsible for TAU hyperphosphorylation, these data can suggest a reduction in the formation of neurofibrillary tangles, which is usually induced by STZ^24^.

Finally, expression of synaptophysin, a biomarker of synaptic integrity was evaluated. The experimental groups did not present statistical changes on the levels of synaptophysin, indicated by the absence of STZ x QTC-4-MeOBnE interaction revealed by two-way ANOVA in both PFC [F_(1,16)_ = 0.06866, p = 0.080] and HC [F_(1,16)_ = 1.584, p = 0.2262] (Fig. 5c,d).

### Toxicological analyses

For toxicological determination, we performed a different experiment, in which mice were divided into three groups 1) canola oil, 2) QTC-4-MeOBnE 0.1 mg/kg and 3) 1 mg/kg treated for 20 days. No rate of morbidity and no toxicological manifestations in weight, skin, fur, eyes, autonomic and central nervous system (CNS) were observed.

As shown in Table 1, there were no changes in hepatotoxicity biomarkers AST and ALT in animals treated with QTC-4-MeOBnE compared to the control. In addition, the chronic compound treatment did not alter the renal biomarkers as creatinine and urea, suggesting the absence of toxicology in QTC-4-MeOBnE treatment for 20 days in these toxicological parameters.

**Table 1:** Effect of sub-chronic administration of QTC-4-MeOBnE (0.1 and 1 mg/kg) for 20 days in toxicological parameters in mice.

	Groups		
	Control	QTC-4-MeOBnE 0.1 mg/kg	QTC-4-MeOBnE 1 mg/kg
AST (U/l)	109.20 ± 15.81	120.21 ± 15.54	110.30 ± 10.27
ALT (U/l)	41.00 ± 3.78	34.20 ± 0.48	41.00 ± 3.78
Urea (mg/dl)	52.08 ± 5.03	44.01 ± 2.83	57.65 ± 4.38
Creatinine (mg/dl)	1.03 ± 0.11	1.01 ± 0.06	1.05 ± 0.07

ALT: alanine aminotransferase; AST: aspartate aminotransferase.

## Discussion

In the present study, we performed a rational design of an MTDL drug based on a virtual screening, exploring the biological targets as AChE, BACE-1 and GSK3- β by molecular docking. The chosen compound, QTC-4-MeOBnE also demonstrated a favorable predicted pharmacokinetics profile and when tested *in vitro*, inhibited the activity of AChE and protected against lipid peroxidation induced by sodium nitroprusside and Fe(II)/EDTA in mice cortex and HP (data not shown). This evidence encouraged us to test the *in vivo* activity of QTC-4-MeOBnE in a model of dementia induced by STZ.

In this sense, we demonstrated the chronic administration of QTC-4-MeOBnE (0.1 and 1 mg/kg) protected against STZ induced cognitive deficit in mice, through different behavioral tests: ORT, Y-maze, SDPA and SRT. In addition, QTC-4-MeOBnA was also able to protect from STZ induced oxidative damage and up-regulation of AChE activity, amyloid cascade and GSK3- β expression. It is worth highlighting that no changes in renal and hepatotoxicity parameters were observed, defining in the first instance, the absence of hepatic and renal toxicity in QTC-4-MeOBnE administration for 20 days.

Recently, the discovery of drugs for AD has gradually tended towards the development of MTDL drugs. The discovery of molecules which can modulate multiple pathways of the disease should significantly advance therapeutic strategies. In summary, based on our prior virtual screening, we have attempted to design a low molecular weight compound which interacts with several promising AD targets. These findings provide support for the translational value of MTDL directly modulating a wide range of therapeutic targets as BACE, GSK-3β and AChE with a possibly interesting pharmacokinetics profile. This suggests that this action mechanism may possess a disease modifying potential for AD.

Consistent evidence has shown that in the majority of cases, clinical manifestations of AD start 10–15 years after the neuropathology began, which makes the prevention of AD characteristics, as induced by STZ, a significant finding. Other research studies have already demonstrated the efficacy of different MTDL’s *in vivo* in AD but with different action mechanisms such as CHF5074 (anti-inflammatory and γ-secretase inhibitor)^25^, ARN14140 (NDMA and AChE)^26^; MT-031 (MAO-A and AChE/BuChE inhibitor)^27^; M30 (propargylamine and chelating)^28^.

Nevertheless, the combination of two or several structural features with specific single-target activity into one structure, face a major challenge related to the structure-activity relationships (SAR) which sometimes makes it difficult to link together distinct pharmacophore groups without losing their associated functionalities^29,30^. In this context, virtual screening and molecular docking might be useful to reshape drug design strategies, to counter determinant steps of the neurotoxic cascade. Thus, the present study shows for the first time, the biological activity of a rational designed moiety, exploring BACE-1, GSK-3β, AChE and oxidative stress therapeutic targets. Although preliminary, these results indicate an interesting new direction for the search for multi-target-directed ligands against AD, as well, the chemical scaffold obtained might be useful for the design of more powerful drugs to overcome the limitations of current single-target drugs in a multifaceted disease.

When STZ is administered by ICV, it decreases cerebral glucose uptake, inducing hypometabolism, accompanied by pathological alterations close to AD, being considered a non-transgenic model of this disease, simulating sporadic AD-like pathology. These alterations include neuroinflammation, metabolic deregulation, cholinergic deficits, accumulation of β-amyloid and tau proteins, and oxidative stress as well as memory and learning impairment^31^. However, besides the STZ insulin resistant state of the brain, a mechanistic explanation for the STZ mode of action and its relation to AD is currently lacking^32^.

In our study two injections of STZ (3 mg/kg) by ICV route unilaterally significantly produced memory impairment linked to AD susceptibility without significant effects on blood glucose levels. The memory impairment was assessed by different behavioral tests trying to mimic different types of memory which are formed throughout life.

Thus, the short-term and long-term memory were assessed by ORT. This test is primarily based on visual recognition to acquisition of memory, based upon the preference of interacting with novel targets among familiar ones^33^. In this study, Y-maze test was utilized to evaluate working memory, which also depends on an associative process. However, the memory evaluated in the Y-maze test also implicates the spatial navigation conditioned to environment^34^. On the other hand, learning mediated aversive memory was evaluated by SDPA, where the mouse subjected learns to associate a context (stepping down) with the occurrence of an aversive event (electric shock)^35^.

All these events are strongly dependent on activity in the hippocampus and cortex (perirhinal, entorhinal, and inferior temporal) being influenced by lesions and damage in these cerebral areas^36^. In support of this, the memory impairment observed in this study may be attributed to neurodegeneration caused by the ICV of STZ. In line with this, QTC-4-MeOBnE (0.1 and 1 mg/kg) is successful in preventing cognitive decline, and can be at least, related to its neuroprotective effect, preventing the oxidative damage induced by STZ in mice cortex and hippocampus, inferred by RS formation and MDA levels.

In addition, the protective effect of QTC-4-MeOBnE was further confirmed through gene expression, in which we studied the genes: APP, BACE-1, γ- secretase, GSK3- β and synaptophysin. STZ significantly induced an over expression of amyloid cascade in HC but failed to achieve this goal in PFC. This event could be explained by amyloid deposition occurring initially and more prominent in HC^37^. Mice treated with QTC-4-MeOBnE showed a reduction of APP, BACE-1, γ- secretase expression in HC but in PFC the level of γ- secretase was reduced without reaching a statistically significant lower expression. This tendency might be confirmed with a higher number of animals.

This capacity of QTC-4-MeOBnE to modulate the amyloid cascade might result in the decreased generation of Aβ. In line with this, we also observed a reduction in GSK3- β expression which can suggest a reduction in TAU hyperphosphorylation, but further studies are needed to confirm this hypothesis. The reduction of Aβ and hyperphosphorylated TAU might explain the effect of QTC-4-MeOBnE in down regulating the activity of AChE, increased by STZ. Some studies have already pointed out these events as crucial for up-regulation of AChE activity in AD^23,27^. Unfortunately, the QTC-4-MeOBnE treatment at this dosage and time, was not able to show an AChE inhibitory activity in animals treated with saline, as we expected. These data can be explained by the time between the euthanasia and compound administration or can suggest a higher dosage or administration time in future studies involving QTC-4-MeOBnE and AChE.

Neuronal loss and synaptic disruption have been associated with cognitive decline in AD and many diseases associated with dementia. However, altered levels of synaptophysin, a major and widespread presynaptic protein, have shown that they are controversial in AD^38,39^. Previous studies reported that synaptophysin levels were not correlated with disease duration^40^. Moreover, recently, Poired *et al*.^41^ found that synaptophysin expression was minimally altered by the progression of dementia, and just in severely demented patients. Furthermore, in this study we did not find statistically significant differences in synaptophysin expression in the PFC and HC of mice submitted to infusion of STZ. These results are in agreement with other previous reports utilizing an STZ-induced mouse model of AD, as in Gratuze *et al*.^42^ and Guo *et al*.^24^, in which levels of synaptophysin or PSD-95 remain unchanged after the ICV (3 mg/kg) of STZ. This evidence suggests that the STZ-induced memory impairment might be attributed to loss of functional synapses or changes in the organization and distribution of synaptic networks without causing substantial synapse volume loss^43,44^. On the other hand, Rai *et al*.^45^ and Xu *et al*.^46^, demonstrated that an ICV of STZ caused a reduction of PSD-95 levels, but not of synaptophysin levels, suggesting that STZ causes majority post synaptic neurotoxicity.

Moreover, drug toxicity is the most challenging drug property that characterizes one of the most significant reasons for many drugs failing to be approved to reach the market^47^. It is widely accepted that adverse reactions and toxicity, generally, are directly proportional to drug dosage^48^. QTC-4-MeOBnE demonstrated therapeutic effects at low dosages such as 0.1 and 1 mg/kg daily. These low drug doses can contribute to the absence of alteration in toxicity parameters tested, such as renal and hepatotoxicity. Besides, we also predict QTC-4-MeOBnE pharmacokinetics, which might suggest a good profile of absorption, distribution, metabolization and excretion. However, further experimental studies are needed to determine its pharmacokinetics, safely, and the pharmacodynamics exerted, which are part of our future study prospects.

In conclusion, we identified QTC-4-MeOBnE as an MTDL compound exploring its interaction with BACE-1, GSK-3β and AChE by molecular docking. The preliminary biological evaluation demonstrated the beneficial effect of QTC-4-MeOBnE in preventing cognitive decline, amyloidosis, oxidative stress, up regulation of AChE and GSK3- β of STZ-induced AD mice, without showing hepatic and renal toxicity. Our results emphasize the emerging strategy of MTDLs and contribute to indicating that these compounds can possibly play an important role in multifactorial diseases, representing a potential approach in anti-AD drug discovery. Furthermore, additional studies about the *in vitro* compound effect and biological validation are required to understand the complete mechanism of this compound, and its physiological effects in different AD models.

## Materials and Methods

### *In silico* analysis

#### Virtual screening

The virtual screening was performed with BACE-1 as an initial target using the ZINC database (http://zinc.docking.org/). We established chemical parameters based on drug-like properties, such as molecular weight <500 KDa, Log P, number of hydrogen donors (<5) and acceptors (<10) to avoid low bioavailability, and poor pharmacokinetics^49,50^. The screening led to 100 drug-like molecules, which were downloaded and manually reviewed to identify a sub-set of candidates that share structural and chemical characteristics with those molecules studied by our research group. At the end of this analysis, 79 structures were created, with the possibility of synthesis using a simple and efficient methodology.

#### Molecular docking

The 79 selected molecules were drawn using ChemDraw and their geometry optimized with Avogadro 0.9.4 following the MMFF94 method^51^. The molecular docking simulation was used as a second screening for these molecules utilizing the Autodock Vina software^52^. We used different structures of BACE (PDB: 1SGZ and PDB: 2ZHU) from the Protein Data Bank (PDB) (http://www.pdb.org/). The CHIMERA 1.5.3 software was used to remove molecules, ions, water and to minimize the structure of proteins, using the Gasteiger charges with 500 steps of minimization^53^.

The ligand and enzyme structures were improved using Auto Dock Tools 1.5.4, in which all the rotatable bonds of ligands were freely rotated and the receptors were considered rigid^54^. For docking studies, we used the Auto Dock Vina (version 1.1.1), utilizing a grid box centered on the active site of the enzymes with high resolution, allowing the program to search for additional sites of probable interactions.

Based on promising results obtained, we explored molecular docking on two other important targets: GSK-3β (PDB: 1H8F) and AChE (PDB:1DX6). The evaluation of the interaction of QTC-4-MeOBnE with AChE was explored using a grid box on CAS and one on the PAS.

#### ADMET evaluation

Unfavorable absorption, distribution, metabolism, elimination and toxicity (ADMET) properties were predicted based on ADMET coefficients in the human body. These characteristics were evaluated using the QuikProp 4.4 software and admetSAR online tool (http://lmmd.ecust.edu.cn:8000).

### QTC-4-MeOBnE synthesis

**Figure 6:**
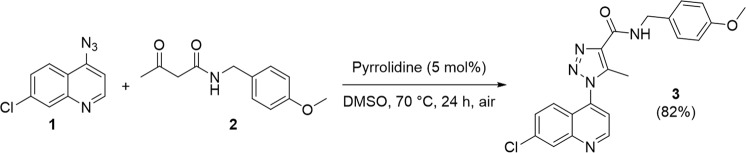
1-(7-chloroquinolin-4-yl)-*N*-(4-methoxybenzyl)-5-methyl-1*H*-1,2,3-triazole-4-carboxamide (QTC-4-MeOBnE) synthesis.

The synthesis of QTC-4-MeOBnE was performed according to our previous protocol using enamide-azide cycloaddition^55^. Thus, to a solution of 4-azido-7-chloroquinoline 1 (1.0 mmol) in DMSO (2.0 mL), was first added the *N*-(4-methoxybenzyl)-3-oxobutanamide 2 (1.0 mmol) and then the catalyst pyrrolidine (5 mol%). The reaction mixture was stirred at 70 °C for 24 hours in an open vial. After completion of the reaction, the crude product was purified by column chromatography on silica gel and the desired product 3 was obtained with an 82% yield (Fig. 6). The spectra and NMR can be visualized in a supplementary section.

### Drugs

QTC-4-MeOBnE was dissolved in canola oil and administered intragastrically (i.g.) in mice.

The STZ was purchased from Sigma-Aldrich (St Louis, USA), dissolved in saline 0.9% and administered by ICV injection at a dose of 3 mg/kg, with a limit rate of 3 μl/site.

All other chemicals of analytical grade were obtained from standard commercial suppliers.

### Animals

The experiments were carried out in adult male Swiss mice (35–50 g). They were maintained under standard housing conditions (room temperature at 22–25 °C) with a 12 h light and dark cycle. The food and water were available *ad libitum*. The behavioral tests and biochemical analyses were performed with 6–7 animals in each group and the gene expression was carried out with 5 animals per group. The study was approved by the Committee on the Care and Use of Experimental Animal Resources of the Federal University of Pelotas (number 4344–2015), in accordance with the National Council for Control of Animal Experimentation guidelines.

### ICV administration

The ICV was administered employing a “free-hand” method as described previously^56,57^ using the bregma fissure as a reference point. Briefly, a microsyringe (25 μL, Hamilton) was inserted perpendicularly and following the coordinates: 0.8 mm posterior to the bregma, 1.0 mm lateral to the sagittal suture, and 3.0 mm beneath the surface of the brain. The needle was inserted unilaterally 1 mm into the midline point equidistant from each eye at an equal distance between the eyes and the ears and perpendicular to the plane of the skull.

First, animals were immobilized under light isoflurane anesthesia and and a gauze soaked in 70% ethanol was used for asepsis of the injection site). The injection was given over 30 s, and the needle was kept in place for a further 30 s to avoid reflux of the injected solution. After ICV injection the peripheral glucose levels were measured using a glucose *monitor* (*Accu*-*Chek*® Aviva) (data not shown). During the dissection of the animal brain, the success of the injection was examined macroscopically, discarding animals whose injection occurred in an inappropriate place or caused cerebral hemorrhage (<5%).

### Experimental design

After three days of adaptation to colony room conditions, mice were randomly divided into six groups:

Group 1 (sham): Canola oil + saline

Group 2: QTC-4-MeOBnE 0.1 mg/kg + saline

Group 3: QTC-4-MeOBnE 1 mg/kg + saline

Group 4: Canola oil + STZ

Group 5: QTC-4-MeOBnE 0.1 mg/kg + STZ

Group 6: QTC-4-MeOBnE 1 mg/kg + STZ

**Figure 7:**
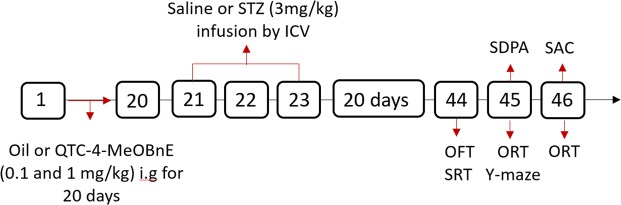
Figure indicating the steps in experimental design. I.g: intragastrically; ICV:intracerebroventricular; OFT: open-field test; ORT: object recognition test; SAC: sacrificed; SPDA: step-down passive avoidance; SRT: social recognition test; STZ:streptozotocin.

Mice were treated with QTC-4-MeOBnE or canola oil i.g for 20 consecutive days. On days 21 and 23, animals received ICV injections of STZ (3 mg/kg) unilaterally, while sham groups were given ICV injections of saline. Twenty days after the second ICV injection, behavioral tests were carried out to evaluate the learning and memory capacity of each group, between days 43 and 45. On day 46, mice were sacrificed, and the brain dissected for further analysis (Fig. 7).

The SDPA was evaluated in a different group of animals, due to the possible alteration caused by the electric shock. In order to reduce the number of animals, the gene expression was evaluated utilizing the highest dose of QTC-4-MeOBnE with its respective vehicles.

### Behavioral tests

#### Open-field test (OFT)

The animals were subjected to OFT for 5 min. The test was conducted in a wooden box (30 × 30 × 15 cm) divided into nine equal squares. The locomotor (scored by the number of segments crossed with the four paws) and exploratory activities (expressed by the number of times the mice stood on their rear limbs) were counted^58^.

#### Object recognition test (ORT)

The object recognition test was performed in an open-field apparatus and conducted according to Rosa *et al*.^59^. Plastic objects with different shapes, composed only by primary colors were placed at the center of the apparatus. One day before the object presentation, each animal was submitted to a habituation session in the absence of objects for 10 min. Firstly, animals were placed in the center of the apparatus containing two identical objects for 5 min as a training phase. After 90 min one object was replaced by another of different form and color, and the exploration of both objects was recorded for 5 min, configuring short-term memory. A second trial was conducted 24 h after the identical object presentation, with another object (different from the first and second in shape and color) comprising the long-term memory.

The results were expressed as exploratory preference according to the following formula: [time spent in the novel object/(time spent in the familiar object + time spent in the novel object)].

#### Y-maze test

The working memory was evaluated according to Sarter *et al*.^60^. Y-maze is a three-arm horizontal maze (40 cm long and 3 cm wide with walls 12 cm high). Mice were initially placed in one arm (A), and the arm entry sequence (e.g. ABCACB, where letters indicate arm codes) and the number of arm entries was recorded for each mouse over a 6 min period.

An alternation was defined as entries into all three arms consecutively (i.e. ABC, BAC or CBA but not BAB) without repetition. The following formula determined percentage alternation: % Alternation = [(Number of alternations × 3)/(Total arm entries − 2)] × 100.

#### Step-down passive avoidance (SDPA)

The non-spatial memory was investigated through the step-down passive inhibitory avoidance task in accordance with Sakagushi *et al*.^61^ with some slight modifications. The apparatus consists of a single box, in which the floor was made of a metal grid connected to a shock scrambler with a safe platform where each animal was placed before starting the experiment. The experiment consists in a training phase and 24 h later, a test session was carried out. The animal was set on the safe platform and when it stepped down with its four paws on the grid floor an electric shock (0.5 mA) was delivered during 2 s. The acquisition memory was defined as 1 min on the safe platform. The test session was performed 24 h later following the same procedure but no shock was given after stepping down.

#### Social recognition test (SRT)

The SRT defined by Kogan *et al*.^62^ was conducted with some modifications. In this test, mice were placed into individual cages immediately prior to the experimental sessions to habituate to the new environment for 5 min. The cages used were identical to those in which the animals were normally housed. After the habituation, a male juvenile mouse (2 weeks old) was placed into the cage with the tested mice for an initial interaction trial, recorded for 5 min. After 90 min, the same procedure was carried out. The social recognition was determined as the time in which the adult spontaneously investigated the younger animal and determined by the index: C_2_/C_1_.

### Biochemical analyses

#### Tissue preparation

On the 46th day, mice were euthanized, and the brain was immediately removed and dissected into prefrontal cortex (PFC) and hippocampus (HC). The PFC and HC were separated into two hemispheres in order to submit each sample to all analyses. The right hemispheres were homogenized in 50 mM Tris-HCl, pH 7.4 (1:4, w/v) to determine reactive species (RS) formation and thiobarbituric acid reactive species (TBARS) levels.

In another group of animals, the right hemispheres were immersed in TRIzol, maintained at −80 °C and submitted to the quantitative real-time polymerase chain reaction (qRT-PCR). Both hemispheres were homogenized in 25 mM sucrose buffer (1:10, w/v) and designated to AChE activity.

The homogenates were centrifuged at 2400 *g* for 10 min to yield a pellet that was discarded and a low-speed supernatant (S_1_) was used for further analysis. The protein concentration was measured by the method of Bradford *et al*. (1976), using bovine serum albumin (1 mg/ml) as the standard.

#### Lipid peroxidation

The lipid peroxidation was evaluated by TBARS formation, as previously described by Ohkawa *et al*.^63^. An aliquot of S_1_ was incubated with 8.1% SDS, 0.8% TBA (pH 3.2) and acetic acid buffer (pH 3.4) at 95 °C for 2 h. Malondialdehyde (MDA), one of the main products of lipid peroxidation, was utilized as biomarker. Sample absorbance was measured at 532 nm, and the results were expressed as nmol MDA/g tissue.

#### Reactive species quantification

The quantification of RS was performed according to Loetchutinat *et al*.^64^. In this assay, an aliquot of S_1_ was incubated with 1 mM dichloro-dihydro-fluorescein diacetate (DCHF-DA) and 10 mM Tris-HCl pH 7.4. The oxidation of DCFH-DA into fluorescent dichlorofluorescein (DCF) is evaluated for intracellular RS detection. The fluorescence intensity was recorded in a spectrofluorophotometer, configured with 520 nm emission and 480 nm excitation and the results were expressed as arbitrary units of fluorescence.

#### AChE activity

AChE activity was carried out according to the method of Ellman *et al*.^65^, using acetylthiocholine as substrate. In this assay the thiocholine (8 mM, acetyl choline analog) complexed to dithiobis-2-nitrobenzoate (DTNB, 10 mM), reduces DTNB to 5-thio-2-nitro-benzoic acid (TNB). TNB has a yellowish color proportional to AChE activity and can be read in the UV light spectrophotometer at 412 nm, at 30 s intervals. The AChE activity was expressed as nmol/*mg protein/min*.

### Gene expression

Total mRNA was extracted in HC and PFC right hemispheres using TRIzol (Invitrogen™, Carlsbad, USA) followed by DNase treatment with DNA-free® kit (Ambion™, USA) and mRNA quantification. The cDNA synthesis was performed using a High Capacity cDNA Reverse Transcription kit (Applied Biosystems™, UK) according to the manufacturer’s protocol.

UltraSYBR Mix (COWIN Bioscience Co., Pequim, China) with the Stratagene Mx3005P was used for amplification. The primers were synthetized by Exxtend (Brasil) and their sequence is described in Table 2. Gene expressions were normalized utilizing β- actin for GSK-3β, APP and γ- Secretase. For BACE-1 and synaptophysin β-microglobulin was utilized as a reference gene. The conditions for the reaction included the cycles: 95 °C for 15 s, 60 °C for 60 s and 72 °C for 30 s. The 2ΔΔCT (Delta–Delta Comparative Threshold) method was used to normalize the fold change in gene expressions.

**Table 2:** Primer sequences utilized in qRT-PCR.

Gene	Primer forward	Primer Reverse
γ- Secretase	TATGGCCTCCTGATTTTTGG	GATGCTAAGCCCTCATCTGC
APP	GCAGTGAGAAGAGTACCAAC	GCAGTGAGAAGAGTACCAAC
BACE-1	CAGTGGAAGGTCCGTTTGTT	GCAGAGTGGCAACATGAAGA
GSK-3β	CGGGACCCAAATGTCAAACT	TCCGAGCATGTGGAGGGATA
Synaptophysin	TGTGTTTGCCTTCCTCTACTC	TCAGTGGCCATCTTCACATC
β2-microglobulin	AAGTATACTCACGCCACCCA	AAGACCAGTCCTTGCTGAAG
β-actin	GTCCCTCACCCTCCCAAAAG	GCTGCCTCAACACCTCAACCC

### Sub-chronic toxicity determination

The toxicity of sub-chronic QTC-4-MeOBnE gavage administration was evaluated in a different experiment. Mice were divided into 3 treatment groups (canola oil, QTC-4-MeOBnE 0.1 mg/kg and 1 mg/kg) which received the corresponding treatment for 20 days. During the treatment, animals were weighed twice a week and observations related to toxicology parameters were done daily. These toxicological parameters include changes in skin, fur, eyes, autonomic (salivation, lacrimation, perspiration, piloerection, urinary incontinence, and defecation), and central nervous system (drowsiness, gait, tremors, and convulsions) changes.

24 h after the last QTC-4-MeOBnE administration, mice were anaesthetized (by inhalation of isoflurane) for blood collection by cardiac puncture. Blood was collected directly in a heparinized tube and centrifuged at 2500 × g for 10 min to obtain the plasma. In view of evaluated biochemical markers of hepatic damage, aspartate (AST) and alanine aminotransferase (ALT) activity were performed, which were expressed as U/l. The renal function was analyzed by determining plasma urea and creatinine levels, which were expressed as mg/dl.

All these parameters were determined by enzymatic colorimetric methods using commercial kits (Labtest Diagnostica, MG, Brazil) according to the manufacturer’s instructions.

### Statistical analyses

All measurements were performed by an independent investigator blinded to the experimental conditions. Data were presented as mean ± standard error of means (S.E.M). Differences among experimental groups were determined by one-way ANOVA for toxicity analyses or two-way ANOVA for the rest of the tests. The post hoc test utilized was Newman-Keuls multiple comparisons tests. All results were analyzed using GraphPad Prism software (version 5.0, Prism software for PC, GraphPad) and were considered statistically significant when P < 0.05.

## Supplementary information

Supplementary infornation
