# Supplementary material for: Rational design, cognition and neuropathology evaluation of QTC-4-MeOBnE in a streptozotocin-induced mouse model of sporadic Alzheimer’s disease
Source: Sci Rep. 2019 May 13;9:7276. doi: 10.1038/s41598-019-43532-9 (PMC6513848; doi:10.1038/s41598-019-43532-9)
Supplement: Supplementary file 1 — Supplementary infornation [file 41598_2019_43532_MOESM1_ESM.docx]

**Rational design, cognition and neuropathology evaluation of QTC-4-MeOBnE in a streptozotocin-induced mouse model of sporadic Alzheimer’s disease**

Mariana G. Fronza*^1^*, Rodolfo Baldinotti*^1^,* Maria Clara Martins^1^, Bruna Goldani^2^, Bianca Thaís Dalberto^2^, Frederico Schmitt Kremer*^3^*, Karine Begnini^4^, Luciano da Silva Pinto*^3^*, Eder João Lenardão^2^, Fabiana K. Seixas^4^, Tiago Collares^4^, Diego Alves^2^ and Lucielli Savegnago*^1^**

^1^ Research Group on Neurobiotechnology - GPN, CDTec, Federal University of Pelotas, UFPel, Pelotas, RS, Brazil.

^2^ Laboratory of Clean Organic Synthesis - LASOL, CCQFA, Federal University of Pelotas, RS, Brazil.

^3^ Laboratory of Bioinformatics and Proteomics - BIOPRO-LAB, CDTec, Federal University of Pelotas, UFPel, Pelotas, RS, Brazil.

^4^ Oncology Research Group - GPO, CDTec, Federal University of Pelotas, UFPel, Pelotas, RS, Brazil.

**Supplementary Information:**

**1-(7-chloroquinolin-4-yl)-N-(4-methoxybenzyl)-5-methyl-1H-1,2,3-triazole-4-carboxamide:** Yield: 0,333 g (82%); Yellow solid; mp 140-141 °C ^1^H NMR (CDCl_3_, 400 MHz): δ 9.13 (d, *J* = 4.5 Hz, 1H), 8.28 (d, *J* = 2.0 Hz, 1H), 7.67-7.64 (m, 1H), 7.57 (dd, *J* = 9.0, 2.1 Hz, 1H), 7.41 (d, *J* = 4.5 Hz, 1H), 7.31 (d, *J* = 8.8 Hz, 3H), 6.90 (d, *J* = 8.8 Hz, 1H), 4.62 (d, *J* = 5.9 Hz, 2H), 3.81 (s, 3H), 2.52 (s, 3H). ^13^C NMR (CDCl_3_, 100 MHz): δ 160.6, 159.0, 151.3, 150.0, 139.6, 138.6, 138.4, 137.1, 130.00, 129.8, 129.2 (2C), 129.0, 123.7, 122.2, 118.7, 114.0 (2C), 55.2, 42.5, 9.30. MS *m/z* (relative intensity): 407 (M^+^, 100), 345 (27), 243 (41), 216 (49), 203 (20), 181 (16), 162 (41).

Spectra:

**Supplementary Figure 1 (S1):** ^1^H NMR (400 MHz) spectrum for compound **3 (**QTC-4-MeOBnE) in CDCl_3_.

**Supplementary Figure 2 (S2)**:  ^13^C NMR (100 MHz) spectrum for compound **3 (**QTC-4-MeOBnE) in CDCl_3_.
